# Cyclosporine A blocks autophagic flux in tubular epithelial cells by impairing TFEB‐mediated lysosomal function

**DOI:** 10.1111/jcmm.16593

**Published:** 2021-05-04

**Authors:** Zhi‐hang Li, Ning An, Xi‐jie Huang, Chen Yang, Hong‐luan Wu, Xiao‐cui Chen, Qing‐jun Pan, Hua‐feng Liu

**Affiliations:** ^1^ Key Laboratory of Prevention and Management of Chronic Kidney Disease of Zhanjiang City Institute of Nephrology Affiliated Hospital of Guangdong Medical University Zhanjiang China

**Keywords:** autophagy, cyclosporine A, lysosome, mTOR, TFEB, tubular epithelial cells

## Abstract

Cyclosporine A (CsA) is an immunosuppressor widely used for the prevention of acute rejection during solid organ transplantation. However, severe nephrotoxicity has substantially limited its long‐term usage. Recently, an impaired autophagy pathway was suggested to be involved in the pathogenesis of chronic CsA nephrotoxicity. However, the underlying mechanisms of CsA‐induced autophagy blockade in tubular cells remain unclear. In the present study, we observed that CsA suppressed the activation and expression of transcription factor EB (TFEB) by increasing the activation of mTOR, in turn promoting lysosomal dysfunction and autophagy flux blockade in tubular epithelial cells (TECs) in vivo and in vitro. Restoration of TFEB activation by Torin1‐mediated mTOR inhibition significantly improved lysosomal function and rescued autophagy pathway activity, suppressing TEC injury. In summary, targeting TFEB‐mediated autophagy flux represents a potential therapeutic strategy for CsA‐induced nephrotoxicity.

## 1 INTRODUCTION

Cyclosporine A (CsA), the backbone of current immunosuppressive regimens, is widely used against the rejection response during solid organ transplantation, dramatically reducing rejection rates and improving early graft survival.^1^, ^2^ However, the chronic administration of CsA is limited by severe nephrotoxicity.^3^ Patients receiving long‐term CsA treatment often experience chronic nephrotoxicity characterized by renal dysfunction, tubular atrophy, tubulointerstitial fibrosis and inflammation.^4^, ^5^ Of note, renal tubular epithelial cells (TECs) are the major targets of CsA‐induced injury.^6^, ^7^ Although a variety of mechanisms have been identified in the initiation and progression of TEC injury due to chronic CsA nephrotoxicity, clinical prevention remains far from satisfactory. Thus, uncovering pathophysiological mechanisms and the development of novel therapeutic strategies for the prevention of CsA‐induced TEC injury are of utmost importance.

Several lines of evidence have demonstrated that autophagy is involved in CsA‐induced TEC injury.^8^, ^9^, ^10^ The activation of autophagy triggered by CsA could decrease misfolded proteins and therefore prevent ER stress‐mediated TEC apoptosis.^8^ In contrast, sustained CsA treatment resulted in excessive autophagosome formation and decreased autophagic clearance in TECs, thus contributing to renal injury.^11^ Autophagy is a multi‐stage dynamic process including initiation, elongation, maturation and degradation. Lysosomal fusion is the terminal process of the autophagy pathway, and its functional integrity is closely associated with the maintenance of autophagic flux. Incomplete maturation and defects in the proteolysis capacity of lysosomes lead to the accumulation of autophagosomes.^12^ Few studies have reported that CsA could cause an impairment of lysosomal structure and proteolytic degradation in renal tubules.^13^, ^14^ Therefore, insufficient lysosomal degradation may contribute to autophagosome accumulation, resulting in TEC injury.

Transcription factor EB (TFEB), a member of the microphthalmia family of basic helix‐loop‐helix‐leucine‐zipper (bHLH‐Zip) transcription factors, is considered a master regulator of lysosomal function and biogenesis, as well as autophagy.^15^, ^16^ In general, phosphorylated TFEB is inactivated and sequestered in the cytosol. Dephosphorylated TFEB is activated and translocates to the nucleus where it directly binds to the CLEAR network element, initiating the transcription of its target genes which encode autophagic and lysosomal proteins.^16^ A decrease in the activation or expression of TFEB has been associated with autophagic flux blockade in several human diseases.^17^, ^18^, ^19^, ^20^

Based on the above findings, we hypothesized that autophagy blockade in CsA‐induced nephrotoxicity may be attributed to the dysregulation of TFEB‐mediated lysosomal function. In the present study, we aimed to test whether restoration of TFEB‐mediated lysosomal function and autophagic flux improves CsA‐induced TEC injury.

## 2 MATERIALS AND METHODS

### 2.1 Animal experiments

The study was approved by the Animal Care and Use Committee of Guangdong Medical University. Eight‐week‐old male BALB/c mice were housed in a temperature‐ and light‐controlled environment. All mice were fed a low‐salt diet (0.01% sodium) and sterilized water. CsA (Selleck, S2286) was diluted in olive oil to a final concentration of 12 mg/mL. The mice were randomly divided into three groups of 10 mice each. In the two CsA‐treated groups, CsA was administered daily by gavage at a dose of 30 mg/kg (low‐concentration group) or 60 mg/kg (high‐concentration group) for 4 weeks. The vehicle‐treated group received daily oral administration of olive oil. After the 4‐week treatment period, mice were killed, and blood and kidney samples were collected for subsequent analyses.

### 2.2 Renal function and histological examination

Serum creatinine levels were measured to assess renal function using Creatinine Assay Kit (Nanjing Jiancheng Bioengineering Institute, China). To evaluate histological tissue injury and tubulointerstitial fibrosis, the mouse kidney samples were fixed in 4% paraformaldehyde and embedded in paraffin; 2‐μm‐thick sections were then prepared and subjected to periodic acid‐Schiff (PAS) or Masson's trichrome staining.

### 2.3 Transmission electron microscopy

Electron microscopy was performed as previously described.^21^ Briefly, kidney tissue specimens from mice were fixed in 0.1 mol/L sodium phosphate buffer containing 2.5% glutaraldehyde. Subsequently, the samples were dehydrated in a graded ethanol series and embedded. Ultrathin sections were stained and visualized with a JEM‐1400 electron microscope (JEOL, Tokyo, Japan).

### 2.4 Immunohistochemistry

Immunohistochemical staining of kidney samples was performed as described previously.^22^ In brief, 2‐μm sections were deparaffinized and then blocked the endogenous peroxidase activity by incubation with 3% H_2_O_2_ at 37°C for 30 min. After blocked the unspecific binding with 3% BSA at room temperature for 30 min, the sections were subsequently incubated with anti‐KIM‐1 (R&D, AF1817), anti‐collagen I (Abcam, ab34710) and anti‐collagen IV (Abcam, ab6586) overnight at 4°C. The sections were washed with PBS three times and then incubated with secondary antibody conjugated with horseradish peroxidase (Beyotime Biotechnology, China) for 1 h at room temperature, subsequently visualized with a colour reagent 3,3‐diaminobenzidine (DAB) (Beyotime Biotechnology, P0203). The KIM‐1–positive tubules, and the collagen I‐ and collagen IV‐positive tubulointerstitium were estimated in at least 20 randomly selected fields (×200).

### 2.5 Cell culture and drug treatment

Human proximal tubular HK‐2 cells (ATCC, CRL‐2190™, Manassas, VA, USA) and mouse renal tubular epithelial cells (mTECs, a gift from Dr Jeffrey B. Kopp, NIH, Bethesda, MD, USA) were cultured as previously described.^23^ To mimic CsA‐induced nephrotoxicity in vitro, the cells were treated with 5, 7.5 and 10 μmol/L CsA (Selleck, S2286) for 24 h or an equal amount of ethyl alcohol in the control group. For pharmacological inhibition of mTOR activity, cells were treated with either 100 nmol/L Torin1 (Selleck, S2827) or DMSO (Sigma, USA) for an additional 6 h after induction of nephrotoxicity via 10 μmol/L CsA. To block the autophagic pathway, the cells were exposed to 10 μmol/L chloroquine (Sigma, C6628).

### 2.6 MTT assays

Cell proliferation was detected using the MTT Cell Proliferation Assay Kit (Biovision, K299). HK‐2 cells were cultured in a 96‐well plate at a density of 5 × 10^3^ cells/well. After experimental treatment, 50 µL of serum‐free medium and 50 µL of MTT reagent were added to each well for 3 h at 37°C. Thereafter, 150 µL of MTT solvent was added to each well and shaken on an orbital shaker for 10 min. A microplate reader (BioTek, ELx800, Winooski, VT, USA) was used to measure the absorbance at 570 nm.

### 2.7 Cell cycle analysis

The cell cycle assay was performed using Cell Cycle and Apoptosis Analysis Kit (Beyotime Biotechnology, C1052) as described previously.^24^ Briefly, HK‐2 cells were harvested and fixed in cold 70% ethanol at 4°C overnight. The cells were washed with cold PBS and then incubated with propidium iodide (PI) staining buffer at 37°C for 30 min in the dark, subsequently analysed by FACS Canto II platform (BD, FACS Canto II, San Jose, CA, USA).

### 2.8 Western blotting

Western blot analysis was performed as previously described.^23^ The membranes were incubated at 4°C overnight with the following primary antibodies: anti‐collagen I (Abcam, ab34710), anti‐TGF‐β1 (Abcam, ab92486), anti‐fibronectin (Abcam, ab23750), anti‐LC3B (Sigma, L7543), anti‐p62/SQSTM1 (Abcam, ab91526), anti‐Cathepsin B (Abcam, ab58802), anti‐phospho‐mTOR (Ser2448) (Cell Signaling, 5536S), anti‐mTOR (Cell Signaling, 2983S), anti‐phospho‐4E‐BP1(Thr37/46) (Cell Signaling, 2855S), anti‐4E‐BP1 (Cell Signaling, 9644S), anti‐phospho‐TFEB (Ser142) (Millipore, ABE1971‐I), anti‐TFEB (BETHYL, A303‐672). β‐actin (Santa Cruz, sc‐47778) and GAPDH (Absin Bioscience, abs132004) were used as the loading control in vivo and in vitro experiments, respectively. The membranes were washed with PBS‐T five times and then incubated with horseradish peroxidase‐labelled secondary antibody (Beyotime Biotechnology, China) for 1 h at room temperature. After visualized with Clarity Max™ Western ECL Blotting Substrates (Bio‐Rad, Hercules, CA, USA), the Azure C500 Western Blot Imaging System (Azure Biosystems, Dublin, CA, USA) was used to detect the immunoreactive signals. The optical density of each band was then quantified using ImageJ software (NIH).

### 2.9 Subcellular fractionation

Nuclear and cytoplasmic fraction was extracted as previously described.^25^ Briefly, cells were suspended in ice‐cold 0.1% CA‐630 (Sigma, I8896) and centrifuged at 4°C, 21 000 *g* for 10 s, and supernatant was collected as cytoplasmic fraction. The corresponding pellets representing the nuclear fractions were washed once and resuspended in ice‐cold 0.1% CA‐630, then sonicated in ice twice for 5 s each. GAPDH (Absin Bioscience, abs132004) and p84 (GeneTex, 5E10) were used as the loading control in cytosolic and nuclear portion, respectively.

### 2.10 Immunofluorescence analysis

Immunofluorescent staining was performed as previously described.^21^, ^23^ Briefly, HK‐2 cells were seeded into coverslips and fixed with 4% paraformaldehyde for 15 min at room temperature, subsequently blocked with 5% BSA for 1 h at room temperature. The cells were then incubated with primary antibodies: mouse anti‐p62/SQSTM1 (Abcam, ab91526) and rabbit anti‐TGF‐β1 (Abcam, ab92486) overnight at 4°C. After washing with PBS, the cells were labelled with Alexa Fluor^®^ 594 donkey anti‐mouse IgG (Invitrogen, A‐21203) and Alexa Fluor^®^ 488 donkey anti‐rabbit IgG (Invitrogen, A‐21206) for 1 h at room temperature. DAPI was used to stain nuclei. Images were obtained using an FV3000 confocal microscope (Olympus, Japan). The puncta/cell for p62 was calculated for at least 30 cultured cells for each group, and the mean fluorescent intensity of TGF‐β1 was quantified using ImageJ software (NIH).

### 2.11 Quantitative real‐time PCR

Total RNA was extracted from cells using RNAiso Plus (Takara, #9109) and reverse transcribed using the PrimeScript RT Reagent Kit (Takara, RR047A). Relative mRNA expression was detected by quantitative real‐time PCR (qPCR) using the TB Green PCR Kit (Takara, RR820A). The primer sequences used were as follows: human *TFEB* forward: 5'‐ACCTGTCCGAGACCTATGGG‐3' and reverse: 5'‐CGTCCAGACGCATAATGTTGTC‐3'; human *β‐actin* forward, 5'‐TCTGGCACCACACCTTCTACAATG‐3' and reverse: 5'‐AGCACAGCCTGGATAGCAACG‐3'.

### 2.12 Ovalbumin dequenching assay

The proteolytic degradation in lysosomes was evaluated using DQ ovalbumin (Invitrogen, D12053) as previously described.^21^, ^23^ For the isolation of primary TECs, kidneys were retrieved and washed with saline. The renal cortices were then minced into tiny particles by cutting with scissors into 1 mL of DMEM medium and subjected to digestion by addition of 10 μL Liberase (Roche, 05401020001) in a water bath at 37°C for 20‐30 min with pipetting every 5 min. Thereafter, 1 mL of DMEM supplemented with 10% FBS was added to stop the digestion and transferred onto a pre‐wetted 70‐μm cell strainer (Biologix, cat# 15‐1070). The filtrate was collected and centrifuged at 200 *g* and 4°C for 5 min. After re‐suspension in 1 mL of DMEM, approximately 2 × 10^5^ primary TECs were added to a six‐well plate and then incubated with 1 µL anti‐Cadherin‐16 antibody (NOVUS, NBP2‐47745) and 2 µg/mL DQ ovalbumin for 2 h at 37°C in the dark. For the in vitro experiment, HK‐2 cells were incubated with 2 µg/mL DQ ovalbumin for 2 h at 37°C in the dark. The mean fluorescent intensity of DQ ovalbumin was quantified by flow cytometry.

### 2.13 LysoTracker Red uptake test

Lysosome acidification was determined using LysoTracker Red (Invitrogen, L7528) as previously described.^21^ Primary TECs isolated from mice and HK‐2 cells were harvested as described above and incubated with 50 nmol/L LysoTracker Red for 30 min at 37°C in the dark. The mean fluorescent intensity of LysoTracker Red was quantified by flow cytometry.

### 2.14 Transfection of lentivirus and plasmid

HK‐2 cells were transfected with flag‐TFEB lentivirus or negative control (GeneChem, Shanghai, China) following the manufacturer's protocol. To assess autophagic flux, HK‐2 cells were transfected with the tandem mRFP‐GFP‐LC3 (tfLC3) plasmid (Addgene, Cambridge, MA, USA) using the Lipofectamine 3000 Transfection Reagent (Invitrogen, L3000015) as previously described.^21^, ^23^ The formation of autophagosomes and autolysosomes was observed in cells after exposure to CsA with or without Torin1 under a FV3000 confocal microscope (Olympus, Japan).

### 2.15 Apoptosis analysis

Apoptosis was determined using an Annexin V‐FITC Apoptosis Detection Kit (Dojindo, Kumamoto, Japan) as previously described.^21^ Briefly, cells were resuspended in 100 μL binding solution containing 5 μL Annexin V‐FITC and 5 μL PI. After incubation at room temperature in the dark for 15 min, another 200 μL of binding buffer was added to a final volume of 300 μL, subsequently analysed by FACScan flow cytometer.

### 2.16 Statistical analyses

All data are expressed as mean ± SEM. Data were analysed by GraphPad with the one‐way analysis of variance (ANOVA) and subsequent Tukey's post hoc test used for comparison of multiple groups or Student's *t* test used for comparisons between two independent groups. A *P*‐value of <0.05 was considered statistically significant.

## 3 RESULTS

### 3.1 CsA causes autophagosome accumulation in TECs

To clarify the role of the autophagy pathway in the pathogenesis of CsA‐induced nephropathy, we established in vivo and in vitro experimental models of CsA‐induced nephrotoxicity. As shown in Figure S1, chronic administration of 60 mg/kg CsA significantly induced TEC injury and tubulointerstitial fibrosis. Similarly to in vivo results, various concentrations of CsA (5, 7.5 and 10 μmol/L) markedly inhibited the proliferation capacity and promoted G1 phase arrest of HK‐2 cells, accompanied by increased expression of extracellular matrix protein (Figure S2).

**FIGURE 1:**
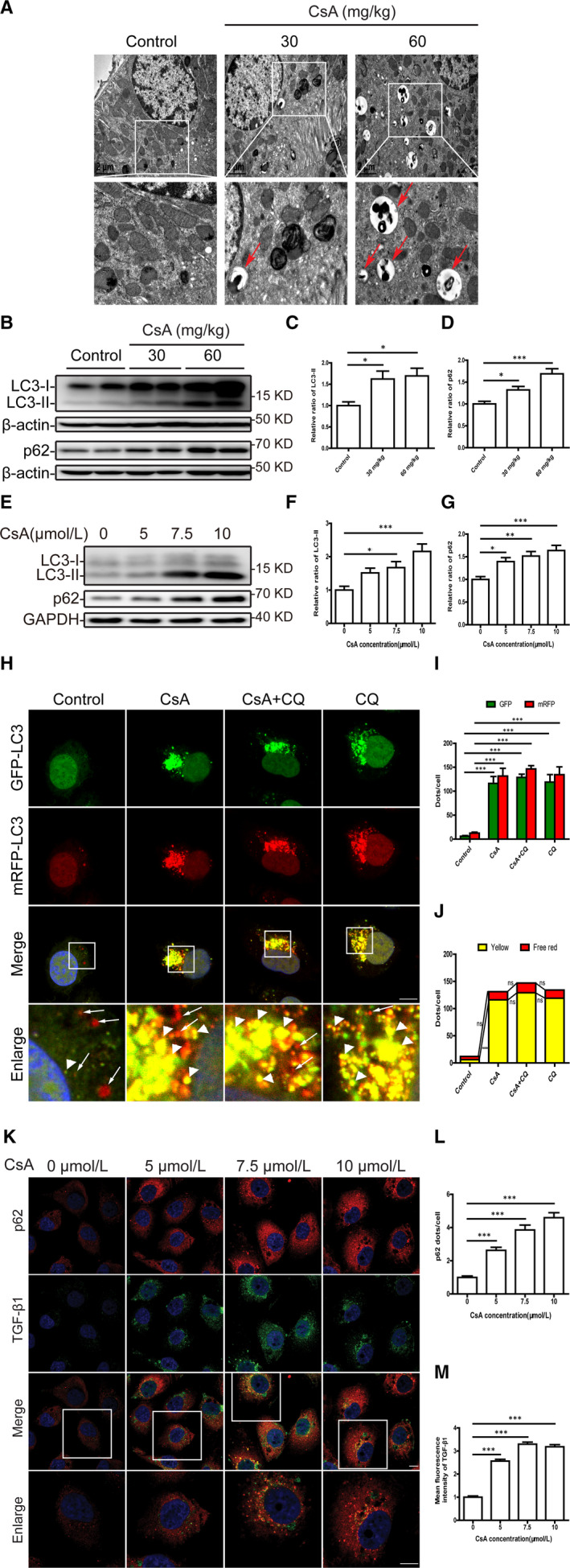
Cyclosporine A induces autophagosome accumulation in TECs in vivo and in vitro. A, Representative transmission electron microscope images of TECs in mouse renal tissue. Red arrow indicates autophagosome accumulation. B‐D, Western blot analysis of LC3 and p62 expression in mouse renal tissue. n = 10 mice per group. E‐G, Western blot analysis of LC3 and p62 expression in HK‐2 cells after exposure to 5, 7.5 and 10 μmol/L CsA for 24 h. H, Representative fluorescence images of autophagic flux in mRFP‐GFP‐LC3 plasmid‐transfected HK‐2 cells after exposure to 10 μmol/L CsA, 10 μmol/L chloroquine (CQ) or CsA plus CQ for 24 h. The yellow puncta indicate autophagosomes (arrowheads), and red puncta indicate autolysosomes (arrows). DAPI was used to stain nuclei. Scale bar: 10 µm. I, Quantitative data for green or red puncta in each cell. J, Quantitative data for yellow puncta or free red puncta in each cell. K, Representative fluorescence images of p62 (red) and TGF‐β1 (green) staining of HK‐2 cells treated with 5, 7.5 and 10 μmol/L CsA for 24 h. DAPI was used to stain nuclei. Scale bar: 10 µm. L, Quantitative analysis of p62 puncta in each cell. M, Quantitative analysis of TGF‐β1 fluorescence intensity in each cell. Each bar represents the mean ± SEM *ns*, no significance. *P < .05, **P < .01 and ***P < .001

We then tested whether the impaired autophagy pathway is associated with TEC injury caused by CsA. Transmission electron microscopy revealed that the TECs of CsA‐treated mice exhibited substantial double‐layer membrane autophagosome formation, which was rare in oil‐treated mice (Figure 1A). Moreover, the protein levels of autophagy‐related markers LC3‐II and p62 were remarkably increased in kidneys from CsA‐treated mice (Figure 1B‐D) and in CsA‐treated HK‐2 cells (Figure 1E‐G), as revealed by Western blotting. Subsequently, to confirm our observation, autophagic flux was further monitored in HK‐2 cells transfected with the tfLC3 plasmid, a construct that distinguishes autophagosomes from autolysosomes based on the greater quenching of GFP in lysosomal acidic environments. In brief, the yellow puncta representing the colocalization of GFP (green) and mRFP (red) signals indicate autophagosomes that are not yet acidified by lysosomes, while free red puncta represent autolysosomes because GFP is quenched whereas mRFP is more stable in acidic environments. As shown in Figure 1H‐J, the amount of autophagosomes (yellow puncta), but not autolysosomes (free red puncta), was significantly increased in CsA‐treated or the lysosomal inhibitor chloroquine (CQ)‐treated HK‐2 cells. Of note, the numbers of yellow and free red puncta were not significantly increased in CsA‐treated cells by addition of CQ compared with the cells treated with CsA only, suggesting CsA results in the disruption of autophagic flux. Immunofluorescence staining also revealed a dose‐dependent accumulation of p62‐positive puncta in the cytoplasm of CsA‐stimulated HK‐2 cells and a simultaneous increase in the expression of profibrotic factor TGF‐β1 (Figure 1K‐M). These findings imply that CsA induces autophagosome accumulation, which may contribute to TEC injury.

### 3.2 CsA impairs lysosomal function in TECs

**FIGURE 2:**
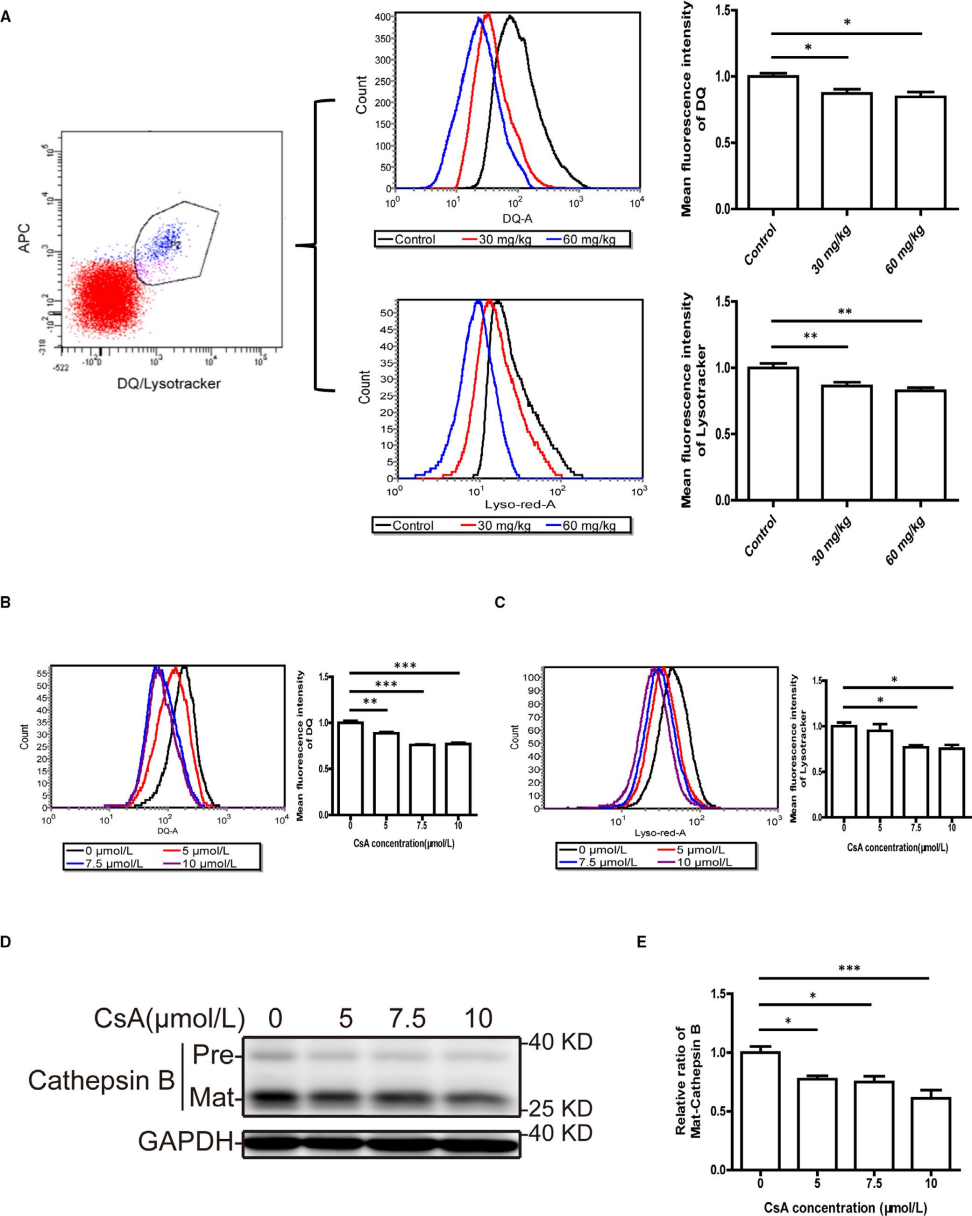
Cyclosporine A impairs the degradation and acidification function of TEC lysosomes in vivo and in vitro. A, Flow cytometric analysis of DQ ovalbumin and LysoTracker Red staining in primary TECs from mice. n = 10 mice per group. B, C, Flow cytometric analysis of DQ ovalbumin and LysoTracker Red staining in HK‐2 cells after exposure to 5, 7.5 and 10 μmol/L CsA for 24 h. D, E, Western blot analysis of cathepsin B expression in HK‐2 cells after exposure to 5, 7.5 and 10 μmol/L CsA for 24 h. Each bar represents the mean ± SEM. *P < .05, **P < .01 and ***P < .001

To determine whether autophagosome accumulation resulted from impaired lysosomal function, we first investigated the efficiency of lysosome‐mediated proteolytic degradation in primary TECs from CsA‐treated mice. DQ ovalbumin was used as a fluorogenic substrate for the detection of lysosomal degradation. When DQ ovalbumin is absorbed and digested by lysosomes, the degradation‐dependent fluorescence signal can be detected by flow cytometry. By utilizing this method, we observed that the fluorescence signal from DQ ovalbumin degradation was markedly suppressed in primary TECs from CsA‐treated mice compared with those from oil‐treated mice (Figure 2A). Consistently, the mean fluorescence intensity of the DQ ovalbumin signal in CsA‐treated HK‐2 cells decreased in a concentration‐dependent manner compared with controls (Figure 2B).

Next, to further evaluate lysosome acidification, which is essential for lysosomal protease activity, LysoTracker Red staining of primary TECs from mice was performed. The mean fluorescence intensity of the LysoTracker Red signal was lower in primary TECs from CsA‐treated mice as detected by flow cytometry (Figure 2A). As shown in Figure 2C, CsA treatment significantly decreased the mean fluorescence intensity of LysoTracker Red in HK‐2 cells as compared to controls. Moreover, Western blotting revealed a dose‐dependent decrease in mature cathepsin B expression in CsA‐treated HK‐2 cells (Figure 2D and E). These results suggested that the degradation function and lysosomal pH in TECs were compromised by CsA treatment.

### 3.3 CsA suppresses lysosomal function by decreasing TFEB activity in TECs

**FIGURE 3:**
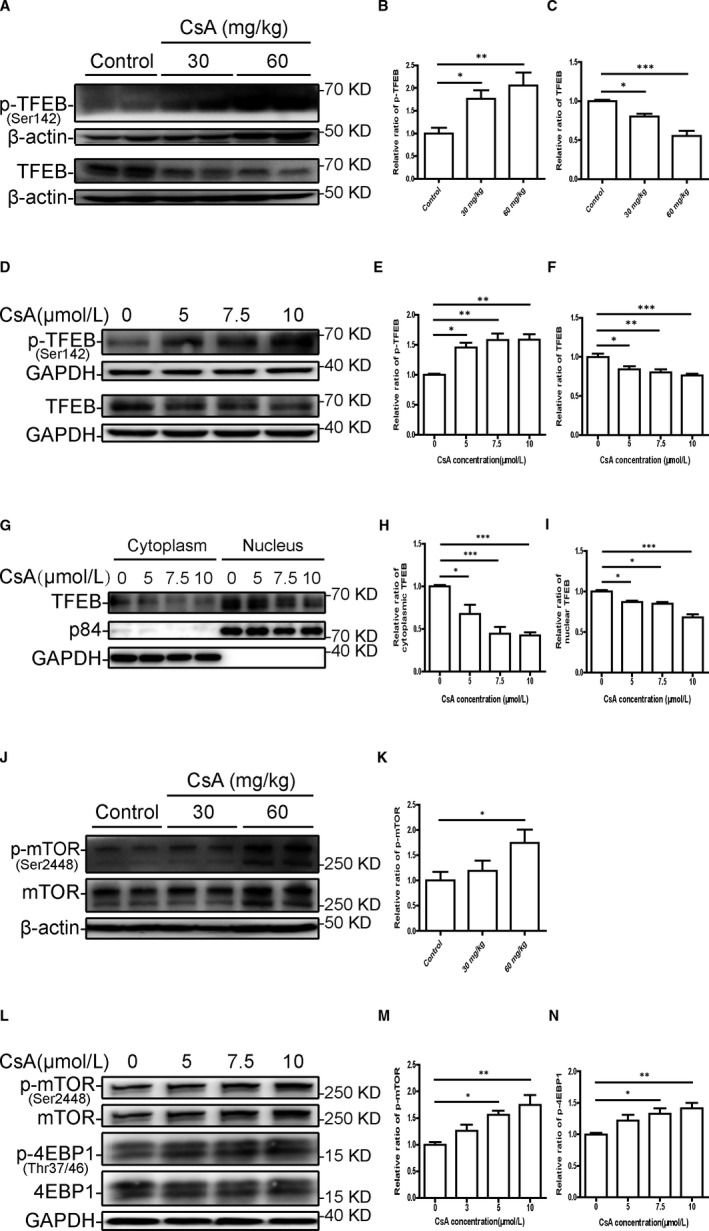
Cyclosporine A induces changes in TFEB phosphorylation and expression by increasing activation of the mTOR pathway. A‐C, Western blot analysis of p‐TFEB (Ser142) and TFEB expression in mouse renal tissue. D‐F, Western blot analysis of p‐TFEB (Ser142) and TFEB expression in HK‐2 cells after exposure to 5, 7.5 and 10 μmol/L CsA for 24 h. G‐I, Western blot analysis of cytosolic and nuclear TFEB expression in HK‐2 cells after exposure to 5, 7.5 and 10 μmol/L CsA for 24 h. J, K, Western blot analysis of p‐mTOR (Ser2448) and mTOR expression in mouse renal tissue. L‐N, Western blot analysis of p‐mTOR (Ser2448), mTOR, p‐4EBP‐1 (Thr37/46) and 4EBP‐1 expression in HK‐2 cells treated with 5, 7.5 and 10 μmol/L CsA for 24 h. Each bar represents the mean ± SEM. *P < .05, **P < .01 and ***P < .001

TFEB is a key transcription factor that regulates lysosome function and maintains autophagic flux. To test whether CsA treatment affects TFEB activation to cause lysosomal dysfunction, we first analysed the expression of TFEB by Western blotting. As shown in Figure 3A‐C, the levels of phosphorylated TFEB at Ser142 (p‐TFEB Ser142 reflects TFEB inactivation) were increased, while total TFEB levels were markedly decreased in kidneys from CsA‐treated mice compared with those from oil‐treated mice. A significant increase in phosphorylated TFEB (Ser142) was also observed in HK‐2 cells after CsA treatment (Figure 3D and E). Furthermore, total TFEB decreased in a concentration‐dependent manner in HK‐2 cells following CsA treatment (Figure 3D and F). In accord with the decreased total TFEB, the cytosolic and nuclear TFEB expression were concentration‐dependent reduced in response to CsA stimulation, as shown by subcellular fractionation analysis (Figure 3G‐I).

Since mTOR is a key regulator of TFEB phosphorylation and ubiquitin system‐dependent degradation, mTOR activity was assessed. Western blotting revealed that 60 mg/kg CsA administration increased renal levels of phosphorylated mTOR (Ser2448) compared with control treatment (Figure 3J and K). A concentration‐dependent increase in phosphorylated mTOR (Ser2448) and its downstream protein 4EBP‐1 (Thr37/46) was also observed in CsA‐treated HK‐2 cells (Figure 3L‐M).

**FIGURE 4:**
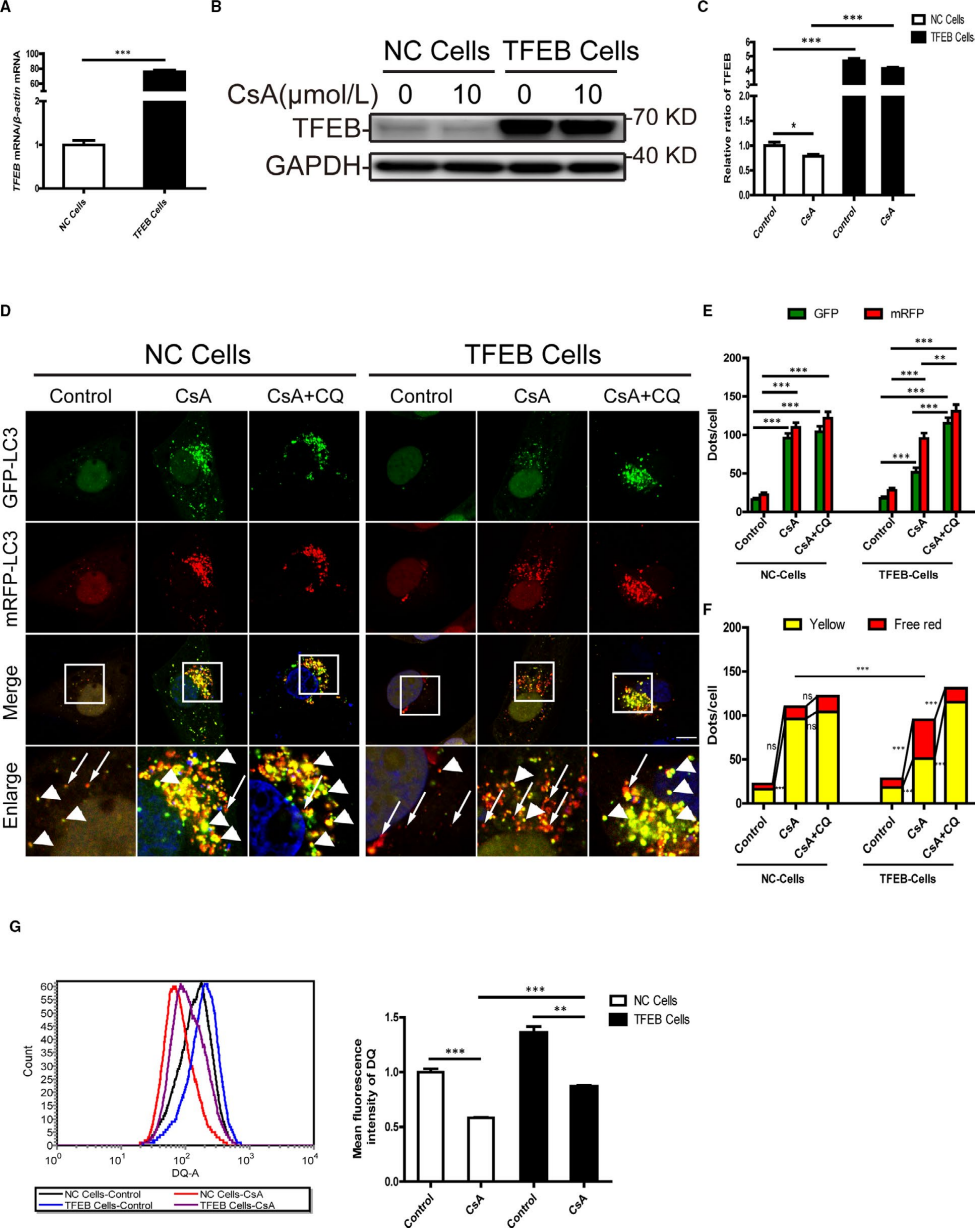
Overexpression of TFEB restores autophagosome clearance and lysosome degradability in cyclosporine A‐treated HK‐2 cells. A, Real‐time PCR analysis of *TFEB* mRNA levels. B, C, Western blot analysis of TFEB expression in negative control cells (NC cells) and stable TFEB‐expressing HK‐2 cells (TFEB cells) after exposure to 10 μmol/L CsA for 24 h. D, Representative fluorescence images of autophagic flux in mRFP‐GFP‐LC3 plasmid‐transfected NC cells and TFEB cells after exposure to 10 μmol/L CsA with or without 10 μmol/L chloroquine (CQ) for 24 h. The yellow puncta indicate autophagosomes (arrowheads), and red puncta indicate autolysosomes (arrows). DAPI was used to stain nuclei. Scale bar: 10 µm. E, Quantitative data for green or red puncta in each cell. F, Quantitative data for yellow puncta or free red puncta in each cell. G, Flow cytometric analysis of DQ ovalbumin staining in NC cells and TFEB cells after exposure to 10 μmol/L CsA for 24 h. Each bar represents the mean ± SEM *ns*, no significance. *P < .05, **P < .01 and ***P < .001

**FIGURE 5:**
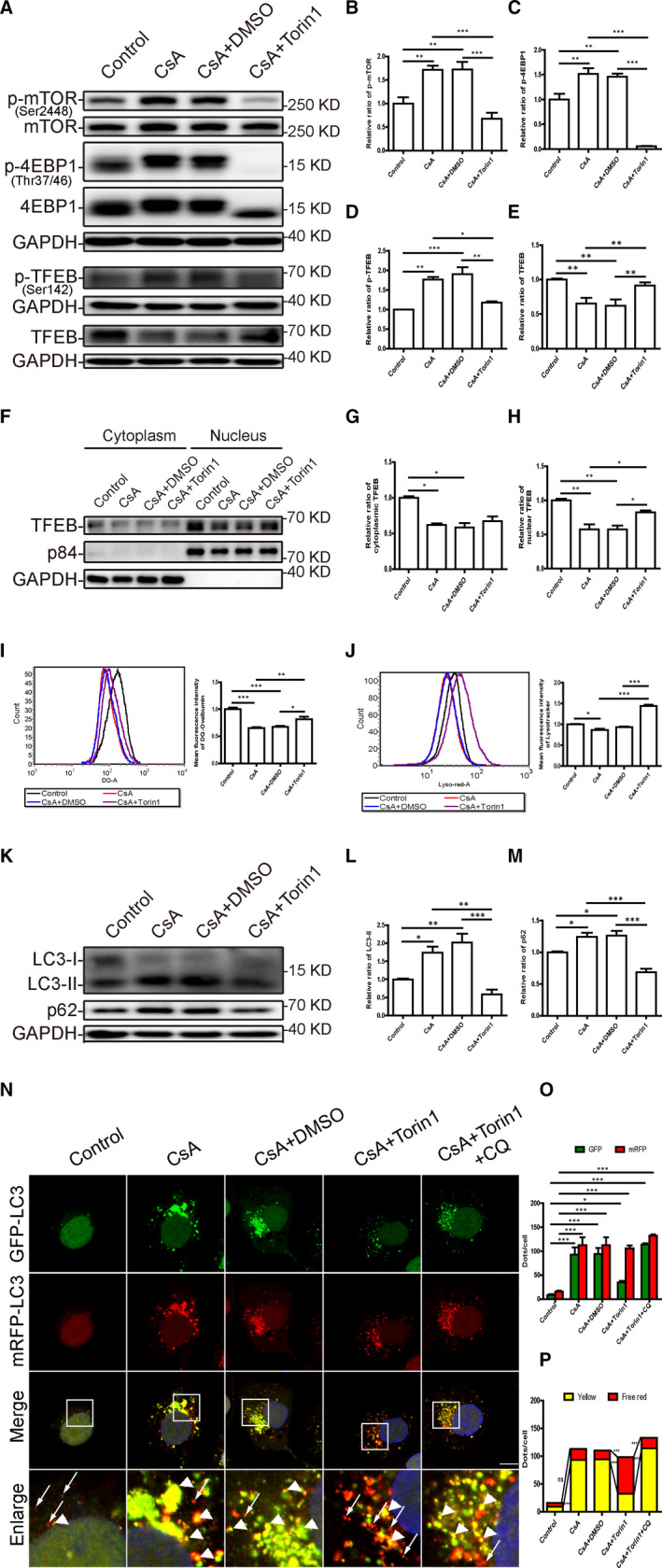
Inhibition of mTOR by Torin1 restores the function of TFEB and improves lysosomal degradation and autophagic flux in cyclosporine A‐treated HK‐2 cells. A‐E, Western blot analysis of p‐mTOR (Ser2448), mTOR, p‐4EBP1 (Thr37/46), 4EBP1, p‐TFEB (Ser142) and TFEB expression in CsA‐treated HK‐2 cells with or without addition of Torin1. F‐H, Western blot analysis of cytosolic and nuclear TFEB expression in CsA‐treated HK‐2 cells with or without addition of Torin1. I, Flow cytometric analysis of DQ ovalbumin staining in HK‐2 cells after exposure to CsA with or without Torin1. J, Flow cytometric analysis of LysoTracker Red staining in HK‐2 cells after exposure to CsA with or without Torin1. K‐M, Western blot analysis of LC3 and p62 expression in CsA‐treated HK‐2 cells with or without addition of Torin1. N, Representative fluorescence images of autophagic flux in RFP‐GFP‐LC3 plasmid‐transfected HK‐2 cells after exposure to CsA with or without Torin1. The yellow puncta indicate autophagosomes (arrowheads), and red puncta indicate autolysosomes (arrows). DAPI was used to stain nuclei. Scale bar: 10 µm. O, Quantitative data for green or red puncta in each cell. P, Quantitative data for yellow puncta or free red puncta in each cell. Each bar represents the mean ± SEM *ns*, no significance. *P < .05, **P < .01 and ***P < .001

To assess the potential effects of TFEB overexpression for improving CsA‐induced autophagy blockade and lysosomal dysfunction, we transduced HK‐2 cells with a lentiviral vector encoding TFEB (hereafter referred to as TFEB cells) and observed robust expression of *TFEB* mRNA (Figure 4A). As shown in Figure 4B and C, CsA treatment significantly reduced TFEB protein levels in negative control cells (hereafter referred to as NC cells), while TFEB levels were significantly higher in TFEB cells. Then, we further investigated the autophagic flux in TFEB cells. In tfLC3‐transfected NC cells, the number of autophagosomes (yellow puncta) was significantly increased under CsA stimulation, which was not significantly changed by CQ addition, indicative of autophagic flux retardation. In contrast, a marked reduction in the number of autophagosomes (yellow puncta) and an increase in the number of autolysosomes (free red puncta) were observed in tfLC3‐transfected TFEB cells after exposure to CsA, which was reversed by the addition of CQ (Figure 5D‐F), indicating TFEB overexpression restores the clearance of autophagosome. Furthermore, the mean fluorescence intensity of the DQ ovalbumin signal was restored in CsA‐treated TFEB cells (Figure 4G). These data suggested that CsA blocked the autophagy‐lysosome pathway by promoting mTOR‐mediated TFEB suppression.

### 3.4 mTOR inhibition by Torin1 restores the function of TFEB, lysosomal degradation and autophagosome clearance in CsA‐treated TECs

To examine whether mTOR inhibition restores the expression and activation of TFEB in CsA‐treated HK‐2 cells and mTECs, mTOR inhibitor Torin1 was used in the following experiments. As shown in Figure 5A‐C and Figure S3C‐E, the phosphorylation of mTOR (Ser2448) and 4EBP‐1 (Thr37/46) was significantly inhibited by the addition of Torin1. Notably, Torin1 treatment markedly decreased the levels of phosphorylated TFEB (Ser142) and restored total TFEB expression (Figure 5A, D and E, Figure S3C, F and G). In parallel, subcellular fractionation analysis showed that Torin1 significantly recovered the nuclear expression of TFEB (Figure 5F‐H).

Flow cytometry revealed that Torin1 significantly enhanced the mean fluorescence intensity of the DQ ovalbumin signal in HK‐2 cells and mTECs treated with CsA (Figure 5I and Figure S3B). Consistently, the mean fluorescence intensity of the LysoTracker Red signal was also markedly increased in CsA‐treated HK‐2 cells with the addition of Torin1 (Figure 5J), indicative of enhanced lysosomal acidification.

Moreover, in CsA‐treated HK‐2 cells and mTECs, Torin1 remarkably suppressed the accumulation of LC3‐II and p62 (Figure 5K‐M and Figure S3C, H and I). Next, autophagic flux was monitored in HK‐2 cells transfected with the tfLC3 plasmid. As shown in Figure 5N‐P, Torin1 significantly increased the number of autolysosomes (free red puncta) and markedly reduced the number of autophagosomes (yellow puncta) in CsA‐treated cells, while CQ compromised the restorative effect of Torin1 on the autophagosome clearance, indicating Torin1 recovers the lysosomal‐dependent autophagosome degradation. These findings demonstrated that restoring the expression and activation of TFEB reverse CsA‐induced autophagy blockade and lysosomal dysfunction in TECs.

### 3.5 Torin1 attenuates CsA‐induced TEC injury and fibrogenesis

**FIGURE 6:**
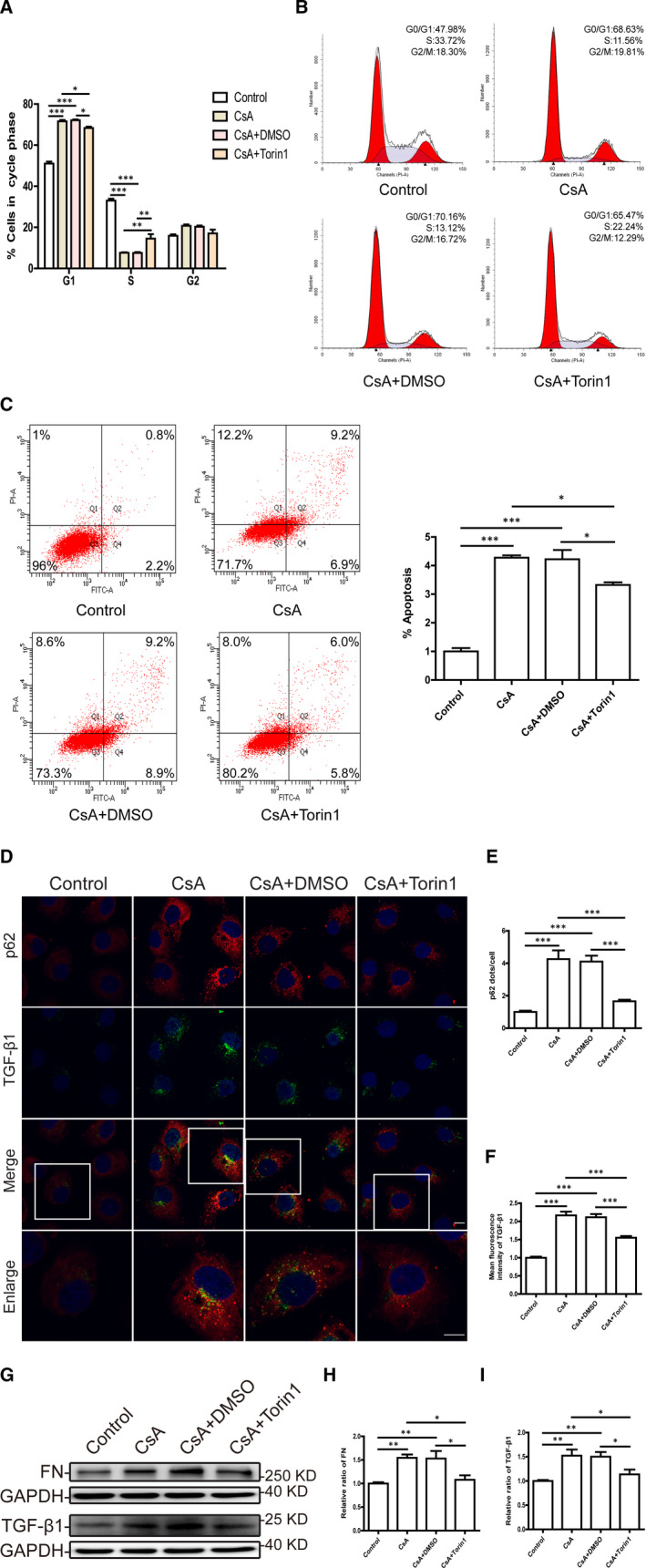
Inhibition of mTOR by Torin1 improves cell injury and fibrogenesis in cyclosporine A‐treated HK‐2 cells. A, Quantitative cell cycle distribution analysis of CsA‐treated HK‐2 cells with or without addition of Torin1. B, Representative images of cell cycle analysis by flow cytometry. C, Flow cytometric analysis of apoptosis in HK‐2 cells after exposure to CsA with or without Torin1. D, Representative fluorescence images of p62 (red) and TGF‐β1 (green) staining in CsA‐treated HK‐2 cells with or without addition of Torin1. DAPI was used to stain nuclei. Scale bar: 10 µm. E, Quantitative analyses of p62 puncta in each cell. F, Quantitative analyses of TGF‐β1 fluorescence intensity in each cell. G‐I, Western blot analysis of FN and TGF‐β1 expression in HK‐2 cells after exposure to CsA with or without Torin1. Each bar represents the mean ± SEM. *P < .05, **P < .01 and ***P < .001

We studied whether CsA‐induced TEC injury could be attenuated after restoring TFEB expression and activation by mTOR inhibition. As shown in Figure 6A and B, the CsA‐induced G1 phase arrest in TECs was partially reversed after Torin1 treatment. Furthermore, a marked decrease in the apoptosis rate of CsA‐treated HK‐2 cells and mTECs was also observed after Torin1 treatment (Figure 6C and Figure S3J). Moreover, immunofluorescence staining indicated that the expression levels of p62 and TGF‐β1 were decreased in CsA‐treated HK‐2 cells by addition of Torin1 (Figure 6D‐F). Western blotting further confirmed that Torin1 reduced the expression of FN and TGF‐β1 in HK‐2 cells and mTECs treated with CsA (Figure 6G‐I and Figure S3K‐M). These data indicated that CsA‐induced injury and fibrogenesis in TECs were alleviated following mTOR inhibition by Torin1.

## 4 DISCUSSION

In the present study, we demonstrated that impaired TFEB function is triggered by mTOR overactivation during CsA‐induced nephrotoxicity both in vivo and in vitro, resulting in lysosomal dysfunction and autophagosome accumulation. Further, restoring the expression and activation of TFEB by mTOR inhibition improved lysosomal function and the clearance of autophagosomes, thus alleviating CsA‐induced TEC injury.

Several studies have reported that autophagy plays a protective role against immunosuppressant‐induced nephrotoxicity.^8^, ^26^ However, CsA‐induced tubular cell cytotoxicity increases once autophagy is inhibited.^8^ Our study confirmed that TEC injury was accompanied by the accumulation of autophagosomes, as indicated by an increase in autophagic vacuole formation marker LC3‐II and the autophagy substrate p62 protein in our CsA‐induced nephrotoxicity model. It was reported that autophagy inhibition enhanced the apoptosis of CsA‐treated TECs,^8^ which is consistent with previous studies on cisplatin nephrotoxicity, obstructive nephropathy as well as ischaemia and reperfusion kidney injury.^27^, ^28^ Interestingly, ginseng extract treatment could attenuate CsA‐induced renal dysfunction, apoptosis and fibrosis by decreasing excessive autophagosome formation and autophagic aggregates.^29^ These findings indicate that autophagy inactivation is one of the important mechanisms underlying the pathogenesis of chronic CsA nephrotoxicity.

A crucial step in the autophagy pathway is autophagosome fusion with the degradative enzyme‐containing lysosome, the function of which is intricately linked with autophagic activity. In the current work, we found that the proteolysis capacity of lysosomes was decreased in primary TECs and HK‐2 cells after treatment with CsA. Consistent with this, a previous report suggested that CsA could impair the activity of the tubular lysosomal proteinase.^14^ Furthermore, transmission electron microscopy revealed lysosomal swelling in TECs from patients receiving CsA treatment.^30^ Most lysosomal enzymes are maintained at an optimal acidic pH, making their function reliant on efficient acidification of the substrate. Inefficient lysosomal acidification often leads to decreased degradation.^31^ As expected, we observed that CsA compromised the acidification of lysosomes in primary TECs and HK‐2 cells, indicating that the decreased proteolysis capacity during CsA‐induced nephrotoxicity results from inefficient lysosomal acidification. More importantly, we and others have reported that defective lysosomes are the main cause of impaired autophagosome clearance in various nephropathies, including diabetic kidney disease, idiopathic membranous nephropathy and urinary protein‐associated disease.^21^, ^32^, ^33^, ^34^, ^35^ These findings suggest that lysosomal dysfunction is the cause of autophagosome accumulation in the CsA‐induced nephrotoxicity model. Therefore, restoring lysosomal function might be a potential therapeutic approach for alleviating CsA‐induced nephrotoxicity.

The molecular mechanisms regulating lysosomal function during autophagy have been extensively studied. A breakthrough discovery was the identification of the Coordinated Lysosomal Expression and Regulation (CLEAR) gene network and its major transcription factor TFEB, which regulates autophagy and lysosomal function.^15^ Several studies have identified TFEB as a therapeutic target for increasing lysosomal activity in lysosomal storage‐associated diseases.^36^, ^37^ However, whether impaired TFEB function is involved in autophagy inhibition and lysosomal dysfunction during CsA‐induced nephrotoxicity has remained unclear. In the present study, we found that CsA treatment not only increased TFEB phosphorylation, but also down‐regulated total TFEB levels in vivo and in vitro. Of note, lysosomal dysfunction triggered by decreased activity and expression of TFEB has been implicated in the pathogenesis of kidney diseases, such as diabetic kidney disease and nephropathic cystinosis,^37^, ^38^, ^39^ as well as various neurodegenerative disorders, such as Alzheimer's disease, Parkinson's disease and Huntington's disease.^17^, ^18^, ^19^, ^20^ Our results demonstrated that overexpression of TFEB restored the proteolysis capacity of lysosomes and decreased the accumulation of autophagosomes during CsA‐induced nephrotoxicity. It has also been reported that TFEB overexpression can suppress the impairment of autophagy in diabetic kidney disease.^39^ Therefore, we propose that dysregulated TFEB function results in lysosome impairment, which is an underlying mechanism leading to autophagy blockade and TEC injury in CsA‐induced nephrotoxicity.

mTOR is the key serine/threonine kinase responsible for the negative regulation of TFEB function by directly phosphorylating TFEB and inhibiting its nuclear translocation as well as by targeting inactive phosphorylated TFEB for degradation via the ubiquitin‐proteasome pathway.^40^, ^41^ We detected the activity of mTOR upon CsA stimulation. Our in vitro and in vivo results revealed a significant increase in phosphorylated mTOR and downstream protein 4EBP‐1, indicating that CsA‐induced TFEB dysfunction may occur in an mTOR‐dependent manner. Indeed, a previous report suggested that long‐term CsA exposure induced up‐regulation of mTOR mRNA and protein levels in the rat kidney.^42^ In addition, evidence from phosphokinase protein array analysis revealed that CsA can increase the phosphorylation of mTOR.^43^ Interestingly, we found that the combination of CsA with Torin1, a typical mTOR inhibitor, largely reversed CsA‐induced lysosomal dysfunction and autophagic blockade by promoting the dephosphorylation and nuclear expression of TFEB. The recovery of lysosomal‐dependent autophagosome degradation was confirmed using the tandem mRFP‐GFP‐LC3 plasmid, as the number of autolysosomes was markedly increased by inhibiting mTOR activity, which indicated that mTOR inhibition could restore TFEB activity to increase lysosomal function and autophagosome degradation. Thus, regulating TFEB function may be a novel therapeutic approach for the prevention of CsA‐induced nephrotoxicity.

It has long been established that CsA causes TEC damage through the mTOR signalling pathway. The present study adds to our understanding of this key pathway, providing new insight into the renoprotective effects of mTOR inhibition against CsA‐induced nephrotoxicity. These effects resulted, at least in part, from the rescue of TFEB function, which enhanced the proteolysis capacity of lysosomes to eliminate accumulated autophagosomes. Based on our findings, it may be reasonable that switch to mTOR inhibitor everolimus and sirolimus in the regimen for kidney transplant recipients in order to reduce chronic transplant dysfunction triggered by chronic CsA nephrotoxicity.^44^, ^45^ However, the complete underlying mechanism remains to be revealed.

**FIGURE 7:**
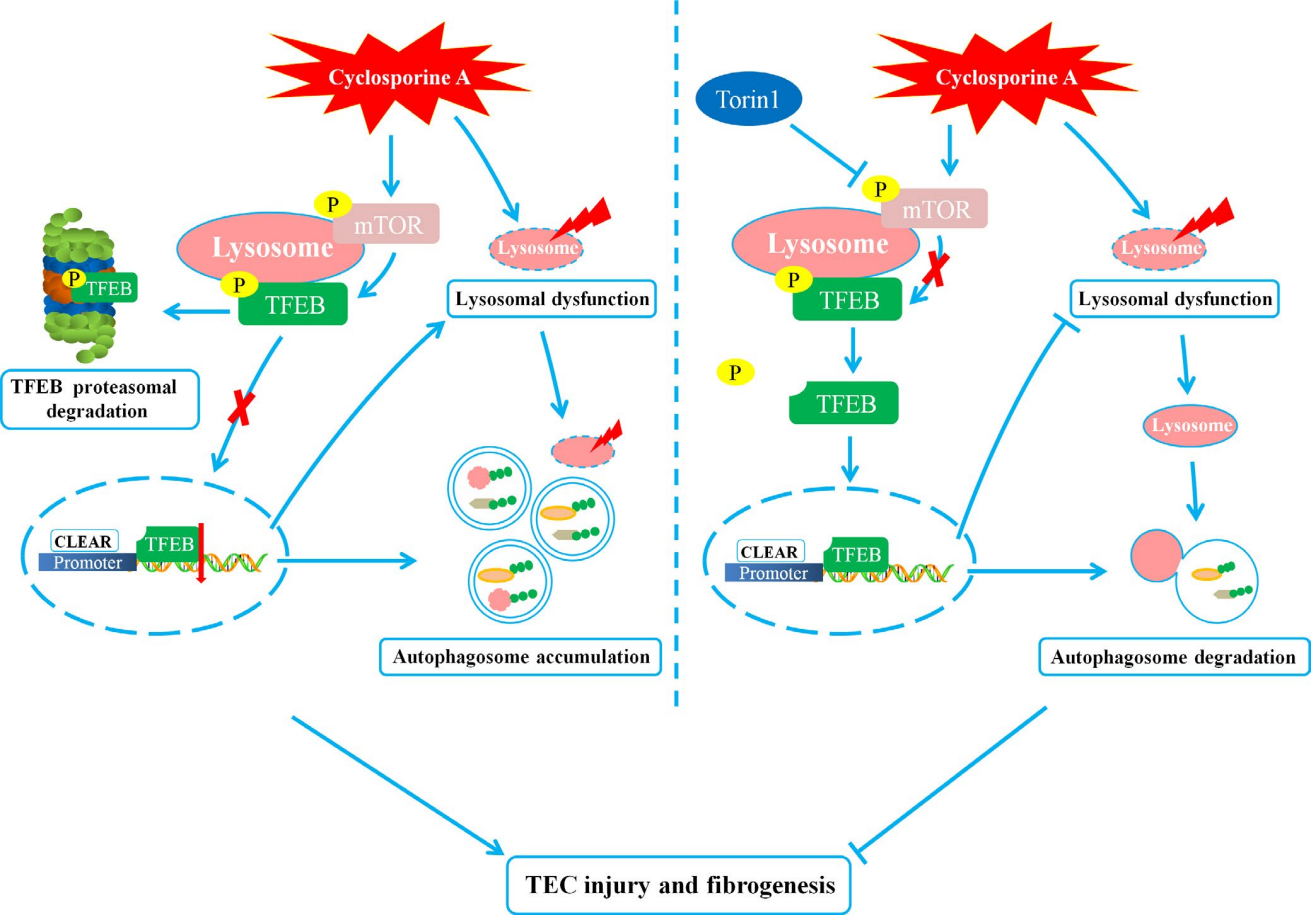
Schematic representation of how the dysregulation of TFEB function contributes to lysosomal dysfunction and autophagy blockade in cyclosporine A‐induced nephrotoxicity. mTOR overactivation induced by CsA up‐regulates TFEB phosphorylation, inhibiting its nuclear translocation, while inactive phosphorylated TFEB is targeted for degradation via the proteasome, resulting in lysosomal dysfunction and autophagy blockade to promote TEC injury and fibrogenesis. Inhibition of mTOR by Torin1 restores the activity of TFEB, leading to an improvement of lysosomal function and autophagosome clearance, in turn alleviating TEC injury and fibrogenesis

In summary, the present study reveals that CsA treatment causes TFEB dysregulation triggered by mTOR overactivation, which results in lysosomal dysfunction and autophagy blockade to promote cellular injury and fibrogenesis. Restoring the activity of TFEB by inhibiting mTOR improved lysosomal function and autophagosome clearance, alleviating TEC injury and fibrogenesis (Figure 7). Our findings advance the understanding of mechanisms underlying the renoprotective effects of mTOR inhibition against CsA‐induced nephrotoxicity and suggest that TFEB might be a potential therapeutic target for the prevention of CsA‐induced nephrotoxicity.

## CONFLICTS OF INTEREST

The authors have no conflicts of interest to declare.

## AUTHOR CONTRIBUTION

**Zhihang Li:** Conceptualization (equal); Data curation (equal); Investigation (equal); Methodology (equal); Software (equal); Writing‐original draft (equal); Writing‐review & editing (equal). **Ning An:** Conceptualization (equal); Funding acquisition (lead); Investigation (equal); Project administration (equal); Supervision (equal); Validation (equal); Writing‐original draft (equal); Writing‐review & editing (equal). **Xijie Huang:** Data curation (equal); Investigation (equal); Methodology (equal); Software (equal). **Chen Yang:** Conceptualization (equal); Methodology (supporting); Writing‐original draft (supporting); Writing‐review & editing (supporting). **Hongluan Wu:** Investigation (supporting); Methodology (supporting). **Xiaocui Chen:** Investigation (supporting); Methodology (supporting). **Qingjun Pan:** Methodology (supporting); Writing‐review & editing (supporting). **Huafeng Liu:** Funding acquisition (lead); Project administration (equal); Supervision (equal); Validation (equal); Writing‐review & editing (equal).

## Supporting information

Fig S1‐S3

## Data Availability

The data that support the findings of this study are available from the corresponding author upon reasonable request.
