# Supplementary material for: Cyclosporine A blocks autophagic flux in tubular epithelial cells by impairing TFEB‐mediated lysosomal function
Source: J Cell Mol Med. 2021 May 4;25(12):5729–43. doi: 10.1111/jcmm.16593 (PMC8184677; doi:10.1111/jcmm.16593)
Supplement: Supplementary file 1 — Fig S1‐S3 [file JCMM-25-5729-s001.pdf]

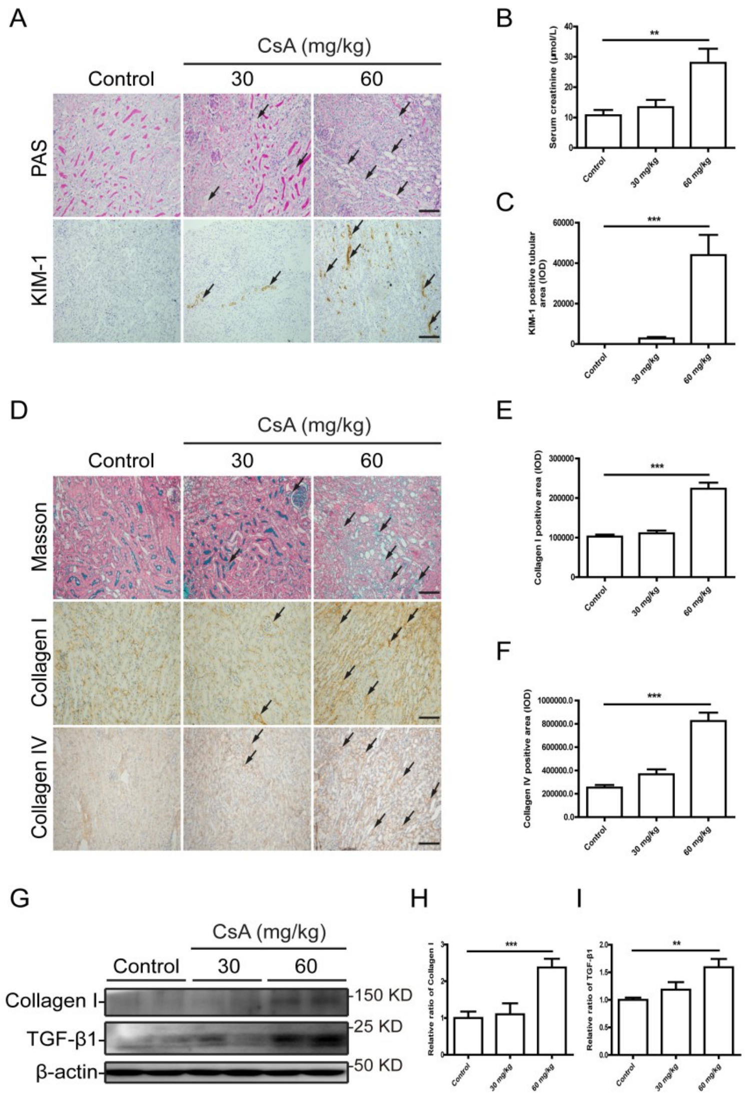

SUPPLEMENTARY FIGURE 1 Cyclosporine A impairs renal function and induces TEC injury and tubulointerstitial fibrosis *in vivo*. (A) Representative kidney tissue sections stained with periodic acid-Schiff (PAS) and KIM-1 (original magnification, ×200). Scale bar: 100 μm. Arrow indicates tubule injury area and positive region of KIM-1 expression. (B) Serum creatinine level was measured in blood samples. (C) Quantitative analyses of KIM-1 expression. (D) Representative kidney tissue sections stained with Masson's trichrome, collagen I and collagen IV (original magnification, ×200). Scale bar: 100 μm. Arrow indicates positive region of tubulointerstitial fibrosis, collagen I and collagen IV expression. (E) Quantitative analyses of collagen I expression. (F) Quantitative analyses of collagen IV expression. (G-I) Western blot analysis of collagen I and TGF-β1 expression in mice renal tissues. Each bar represents the mean ± SEM. **$P < 0.01$ and ***$P < 0.001$. n = 10 mice in each group.

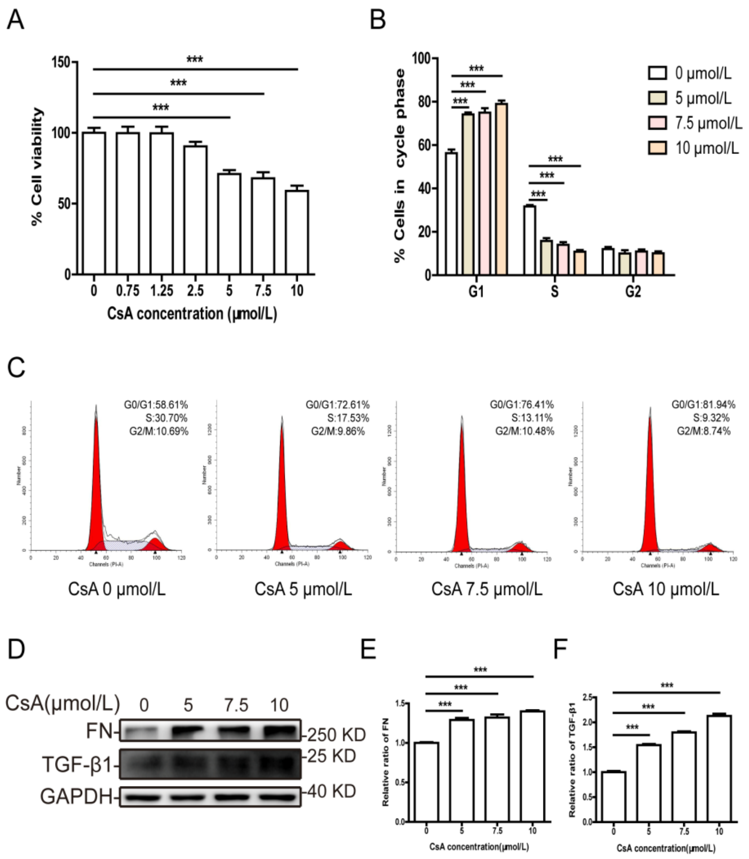

SUPPLEMENTARY FIGURE 2 Cyclosporine A inhibits the proliferation and induces the expression of FN and TGF-β1 in HK-2 cells. (A) MTT assays for the determination of HK-2 cells proliferation capacity after exposure to various concentrations of CsA for 24 h. (B) Quantitative analyses of cell cycle distribution in HK-2 cells treated with 5, 7.5 and 10 μmol/L CsA for 24 h. (C) Representative images of cell cycle analysis by flow cytometry. (D-F) Western blot analysis of FN and TGF-β1 expression in CsA treated HK-2 cells. Each bar represents the mean ± SEM. ***$P < 0.001$.

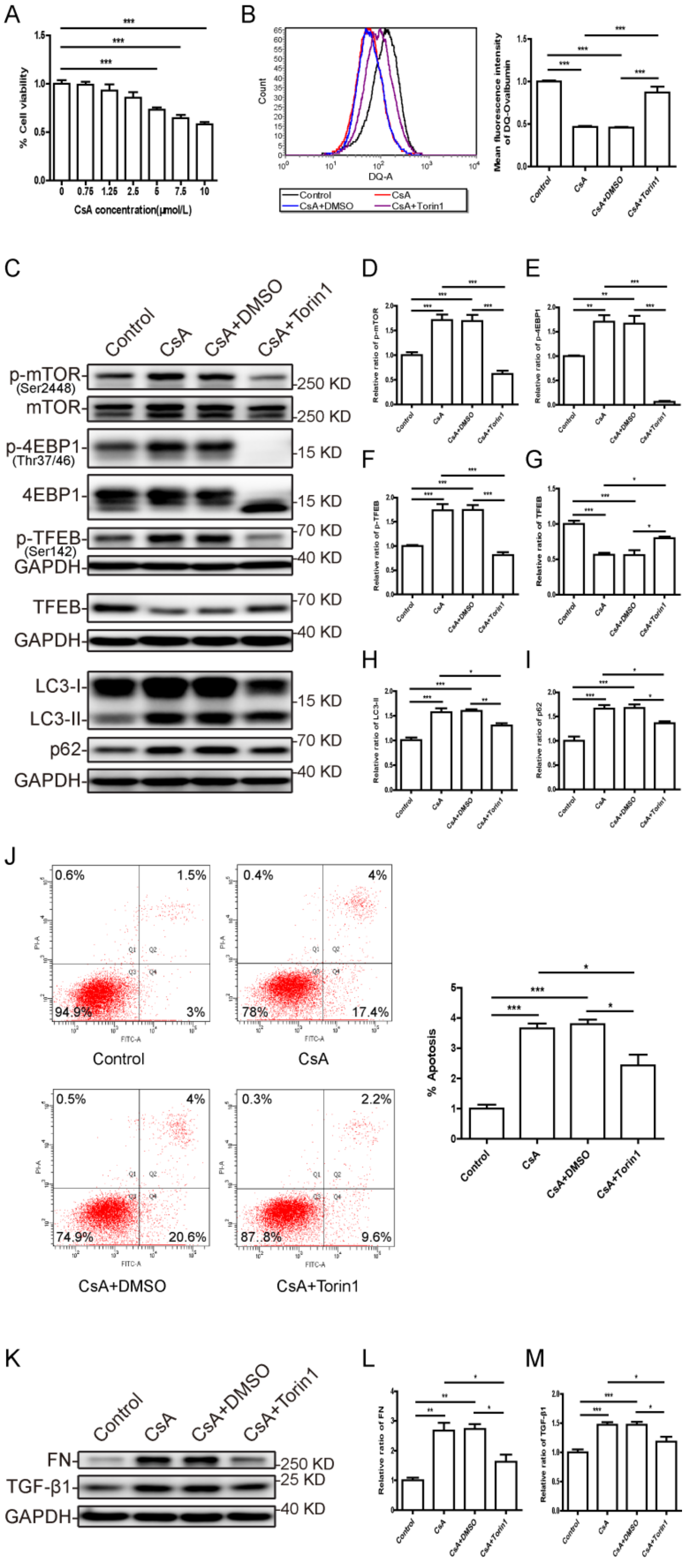

SUPPLEMENTARY FIGURE 3 Inhibition of mTOR by Torin1 restores the function of TFEB and improves lysosomal-dependent autophagosome degradation, cell injury

and fibrogenesis in cyclosporine A-treated mouse renal tubular epithelial cells. (A) MTT assays for the determination of mouse renal tubular epithelial cells (mTECs) proliferation capacity after exposure to various concentrations of CsA for 24 h. (B) Flow cytometric analysis of DQ-ovalbumin staining in mTECs after exposure to CsA with or without Torin1. (C-I) Western blot analysis of p-mTOR (Ser2448), mTOR, p-4EBP1 (Thr37/46), 4EBP1, p-TFEB (Ser142), TFEB, LC3 and p62 expression in CsA-treated mTECs with or without addition of Torin1. (J) Flow cytometric analysis of apoptosis in mTECs after exposure to CsA with or without Torin1. (K-M) Western blot analysis of FN and TGF-β1 expression in mTECs after exposure to CsA with or without Torin1. Each bar represents the mean ± SEM. $^{*}P < 0.05$, $^{**}P < 0.01$, and $^{***}P < 0.001$.
